# LC-MS/MS Determination of Cereulide in Food and Infant Formula: Method Validation and Six-Year Survey in Italy

**DOI:** 10.3390/foods15173129

**Published:** 2026-09-03

**Authors:** Elena Torres, Tabata Bezzo Llufrio, Stefania Massafra, Gian Luca Ferro, Alessio Gasperin, Alessia Ossino, Daniela Manila Bianchi, Maria Cesarina Abete, Marilena Gili

**Affiliations:** Istituto Zooprofilattico Sperimentale Piemonte, Liguria e Valle d’Aosta, Via Bologna 148, 10154 Turin, Italy; elena.torres@izsplv.it (E.T.); stefania.massafra@izsplv.it (S.M.); gianluca.ferro@izsplv.it (G.L.F.); alessio.gasperin@izsplv.it (A.G.); alessia.ossino@izsplv.it (A.O.); manila.bianchi@izsplv.it (D.M.B.); mariacesarina.abete@izsplv.it (M.C.A.); marilena.gili@izsplv.it (M.G.)

**Keywords:** cereulide, LC-MS/MS, foodborne outbreaks

## Abstract

Among pathogenic microbes that can produce toxins, *Bacillus cereus* plays an important role in foodborne disorders. It is responsible for the production of cereulide, a cyclic dodecadepsipeptide toxin, very stable to heat and acidic pH, that can cause emetic syndromes. The study describes the secondary validation of the ISO 18465 method for food matrices in the concentration range 1–100 μg/kg and the in-house validation of a more sensitive liquid chromatography coupled with tandem mass spectrometry (LC-MS/MS) method for infant formula at the lower concentration levels required by the EFSA since 2026. To improve the applicability of cereulide determination in infant formula, the solid matrix was reconstituted before extraction and the analysis was performed on the SCIEX QTRAP 7500 system. The method showed good linearity (0.010–1.000 μg/L, R^2^ = 1000), a limit of quantification equal to the half-maximum level (LM = 0.054 μg/L, LOQ = 0.025 μg/L), good recovery (100–109%) and acceptable within-laboratory reproducibility (RSD% < 15%). This method provides a reliable analytical tool for cereulide monitoring in a broad variety of food commodities not considered before by ISO 18465 and makes it possible to reach the new lower limits established for infant formula. Furthermore, data on 332 samples involved in suspected food poisoning in Italy between 2019 and 2024 are presented in this retrospective study.

## 1. Introduction

Food contamination represents a great threat in the food industry. Despite various efforts to prevent bacteria or fungi contaminations, toxins can resist harsh conditions, leading to foodborne diseases. Bacterial toxins can be resistant to several treatments used in food processing and storage such as ohmic heating, radio frequency and microwave heating, pulsed electric field technology, high-pressure processing, high light intensity technology, ultrasonic techniques and pulsed X-rays [1].

EFSA reports on zoonoses, zoonotic agents and foodborne outbreaks (FBOs) have consistently identified bacterial toxins as a major cause of foodborne disease in Europe. In the 2017 EU summary report, bacterial toxins were already among the most frequently reported causes of FBOs, ranking after Salmonella and other bacterial pathogens [2]. The European Union One Health 2022 Zoonoses Report further highlighted the growing importance of toxin-mediated foodborne illness, identifying bacterial toxins as the second most frequently reported group of causative agents in FBOs and the leading group in terms of associated human cases. This increase was largely attributed to enhanced reporting of outbreaks linked to *Bacillus cereus* toxins [3]. The most recent European Union One Health 2024 Zoonoses Report confirms that bacterial toxins continue to represent a substantial public health burden, remaining the second most frequently reported known causative agent of FBOs in the European Union [4]. In 2024, outbreaks associated with *B. cereus* toxins were among the most commonly reported bacterial toxin outbreaks (1.9% of FBOs) and contributed substantially to the overall number of cases, including several fatal outcomes. Together, these data indicate that while bacterial toxin-associated outbreaks remain persistently important in Europe, the epidemiological relevance of *B. cereus* has increased over recent years, making it one of the principal bacterial toxin producers involved in foodborne disease across the European Union [4].

*B. cereus* is a Gram-positive and rod-shaped bacterium, forming spores resistant to food preparation and cooking and able to germinate and arise different toxins, in particular one emetic exotoxin and three diarrheal enterotoxins [5]. The enterotoxins are produced during vegetative growth of *B. cereus* in the small intestine and are responsible for the diarrheal syndrome of food poisoning [6].

**Figure 1:**
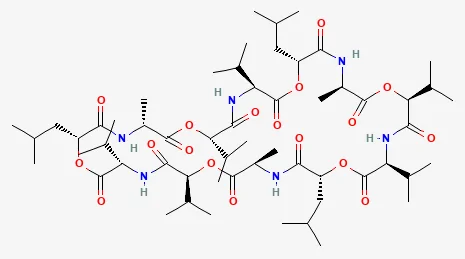
Chemical structure of cereulide, a cyclic dodecadepsipeptide produced by emetic strains of Bacillus cereus. Reproduced from PubChem Compound Summary for Cereulide (CID 6441099) [11].

Instead, the emetic syndrome is caused by a small cyclic polypeptide pre-formed in food, encoded by non-ribosomal peptide synthetase genes (*ces*) and called cereulide [7,8]. Cereulide is a heat-stable cyclic dodecadepsipeptide (Figure 1) [9], resistant to heating at 150 °C and not inactivated in the gastro-enteric tract by very acidic pH and protease [10].

Usually, the ingestion of the pre-formed toxin in food by *ces*-positive *B. cereus* strains is responsible for emetic syndromes. Symptoms include nausea and vomiting, one to six hours after ingestion of contaminated food and finish after one day. Even if effects of intoxication are often mild, occasionally they implicate severe clinical manifestations including acute liver failures or even death [12,13,14,15].

Emetic *Bacillus cereus* food poisoning is typically associated with foods containing high numbers of cereulide-producing strains (commonly 10^5^–10^8^ CFU/g) [16], although disease severity depends primarily on the amount of pre-formed cereulide present in the food. Fatal cases and outbreaks have been linked to foods containing approximately 10^7^–10^8^ CFU/g *B. cereus*. [17,18].

Toxin synthesis occurs only under certain conditions of pH and temperature [19,20] and is favored by a high starch content, so foods most often involved in cereulide poisoning are represented by preparations based on rice, pasta and potatoes [21,22].

In recent years, however, growing attention has been directed toward the potential contamination of infant formula, a critical food source for a highly vulnerable population. Between late 2025 and early 2026, several precautionary recalls and withdrawals of infant and follow-on formula products were issued across Europe following the detection of cereulide in specific batches. Evidence from these events suggested that contamination may originate from lipid-rich ingredients, particularly arachidonic acid (ARA) oils used in formula fortification, consistent with the strong lipophilicity of cereulide and its tendency to accumulate in fat-containing matrices [18,23].

The relevance of cereulide in infant nutrition is amplified by several factors. Infants consume large volumes of formula relative to their body weight, often as their sole source of nutrition, which increases potential exposure. In addition, infant physiology—characterized by immature detoxification systems, developing mitochondria, and reduced renal clearance—may enhance susceptibility to the toxin’s mitochondrial effects and prolong systemic exposure [24,25]. These considerations have prompted regulatory authorities, including the EFSA, to derive an acute reference dose (ARfD) for cereulide in infants (0.014 µg/kg body weight), reflecting a precautionary approach in the context of limited toxicological data [23]. The rapid risk assessment conducted by the EFSA established that cereulide concentrations above 0.054 μg/L in newborn formulae and 0.1 μg/L in formulae after 6 months may result in the exceeding of the derived acute reference dose (ARfD) in infants.

Several analytical methods have been developed for cereulide detection and quantification in food. Some approaches are non-specific bioassays based on boar spermatozoan motility inhibition [26] or on HEp-2 cell vacuolization [27]; however, they can give only an approximate toxicity titer, but not an accurate concentration. Concerning chemical assays for toxin quantification, methods are based on liquid chromatography coupled with mass spectrometry (LC-MS) [28,29,30,31]. These techniques exploit high-performance LC (HPLC) coupled to ion trap MS, which enables very accurate detection of low cereulide concentrations. Moreover, these methods often exploit valinomycin as the internal standard, whose MS response is significantly influenced by the food matrix [29,30]. Bauer et al. 2010 overcome this drawback introducing a stable isotope dilution analysis (SIDA) with ^13^C_6_-cereulide as the internal standard [32,33].

This approach was further developed in ISO 18465:2017 [34], which describes an LC-MS/MS method for cereulide quantification using ^13^C_6_-cereulide as an internal standard. The method enables the identification and quantification of cereulide with high sensitivity and specificity (LOQ = 1 μg/kg).

However, the scope of ISO 18465:2017 is limited to starch-rich foods, such as rice, pasta, and potatoes, and does not include other food matrices frequently implicated in foodborne outbreaks and commonly analyzed within official control activities. Furthermore, the method’s limit of quantification (LOQ) is not sufficiently low to allow reliable quantitative determination of cereulide at the concentration levels required for infant formula, as recently established by the EFSA.

In infant formula, strong matrix effects and incomplete release of cereulide from lipid-protein complexes or encapsulated ingredients can reduce analytical accuracy and reproducibility. To address these limitations, recent methodological developments have focused on matrix-specific optimization, such as hydration-assisted extraction combined with solid-phase extraction (SPE) clean-up and ultra-performance LC–MS/MS analysis, which improves analyte recovery and reduces interference [35].

However, this approach remains time-consuming, costly, and analytically labor-intensive, which may limit its suitability for routine large-scale monitoring and official control activities.

The aim of this study is the evaluation of EN-ISO 18465:2017 applicability to a broad variety of food commodities not considered before in the ISO and the in-house validation of a quantitative method for infant formula at the lower limits established by EFSA risk assessment. Finally, the results of a six-year control carried out analyzing 332 food samples involved in suspected food poisoning are discussed.

## 2. Materials and Methods

### 2.1. Reagents, Standards and Test Materials

Acetonitrile, methanol, n-heptane, formic acid and ammonium formate, HPLC- or MS analytical-grade, were purchased by Merck (Milan, Italy).

Blank control matrix samples (cooked rice) were included in each analytical batch and were verified to be free of endogenous cereulide prior to use. These blank matrices were then used for the preparation of negative controls and spiked quality control (QC) samples to assess method performance.

Certified Reference Material (CRM) cooked rice with a certified value of cereulide at 11.3 ± 0.8 μg/kg was purchased from JRC Geel, Belgium.

Cereulide standard stock solution (50 ng/μL in acetonitrile) and labeled internal standard ^13^C_6_-cereulide stock solution (20 ng/μL in acetonitrile), of certified purity were purchased by Chiralix (Nijmegen, The Netherlands).

From these stock solutions, the intermediate standard solutions of cereulide and ^13^C_6_-cereulide were prepared at the concentration levels listed in Table 1 by dilution in acetonitrile. All solutions are stable at −20 °C for at least 3 years.

The calibration standard solutions of cereulide and ^13^C_6_-cereulide for food analysis were prepared at the concentration levels listed in Table 2 directly in vial.

The calibration standard solutions of cereulide and ^13^C_6_-cereulide for infant formula analysis were prepared at the concentration levels listed in Table 3 by dilution in acetonitrile in volumetric flask. All solutions are stable at −20 °C for at least 6 months.

### 2.2. Food Sample Collection

Meals involved in suspected food poisoning were collected by the veterinary services of local health authorities or by food inspection services. The samples were delivered to the laboratories at Istituto Zooprofilattico Sperimentale del Piemonte, Liguria e Valle d’Aosta (IZSPLV) to determine the presence of *B. cereus* containing *ces*-genes and to quantify the cereulide toxin. In total, 332 samples involved in suspected foodborne outbreaks were analyzed for the emetic toxin in the period 2019–2024. All samples were frozen and stored at −20 °C until analysis.

### 2.3. Sample Preparation

#### 2.3.1. Food

An amount of 2.5 g of homogenized sample was spiked with 500 μL of ^13^C_6_-cereulide–C solution (0.1 ng/μL), obtaining a corresponding concentration in matrix of 20 μg/kg. The analyte was extracted with 30 mL of acetonitrile, by Ultra-Turrax (IKA-Werke GmbH & Co. KG, Staufen im Breisgau, Germany) homogenization followed by 1 h of agitation on a mechanical shaker. After centrifugation (15 min, 4000 rpm), 5 mL of supernatant was defatted twice with 3 mL of n-heptane. Purified extract was filtered on a 0.2 µm polypropylene filter before injection into LC-MS/MS.

#### 2.3.2. Infant Formula

For infant formula powders, reconstitution was performed at 40 °C following the instructions given by producer or using a powder-to-water ratio of 1:8 when not specified. Then, 3 mL of liquid or reconstituted sample was spiked with 75 μL of the ^13^C_6_-cereulide–D solution (0.010 ng/μL), obtaining a corresponding matrix concentration of 0.250 μg/L. The analyte was extracted with 27 mL of acetonitrile by 1 h of agitation on a mechanical shaker. After centrifugation (15 min, 4000 rpm), 5 mL of supernatant was defatted twice with 3 mL of n-heptane. Purified extract was filtered on a 0.2 µm polypropylene filter before injection into LC-MS/MS.

### 2.4. LC-MS/MS Analysis

LC-MS/MS analysis was carried out on three different analytical systems: Dionex UltiMate 3000 Stack HPLC System (Thermo Fisher Scientific, Germering, Germany) coupled to an MS/MS spectrometer TSQ ENDURA (Thermo Fisher Scientific, San José, CA, USA), UHPLC EXION coupled to an MS/MS spectrometer QTRAP 6500+ (AB SCIEX, Marlborough, MA, USA) and UHPLC 1290 Infinity (Agilent Technologies, St. Clara, CA, USA) coupled to an MS/MS spectrometer QTRAP 7500 (AB SCIEX, Marlborough, MA, USA), all equipped with a H-ESI running in positive and negative mode and operating in multiple reaction monitoring (MRM) mode.

Chromatographic separation was performed on an X-Select HSST3 column^®^ (100 × 3.0 mm, 2.5 µm) analytical column equipped with an X-Select HSST3 (5 × 2.1 mm, 2.5 μm) column guard purchased from Waters, Milford, MA, USA.

The LC eluents were: 0.1% formic acid in methanol (B) and 10 mM ammonium formate in water containing 0.1% formic acid (A). The gradient started at 90% B for 0.5 min, linearly increased to 100% over 3.5 min and kept at 100% for 5 min, then reduced to 90% over 2 min, following an equilibration period of 4 min. The injection volume was 10 μL. The autosampler temperature was 15 °C and the oven temperature was 45 °C.

Separation was obtained using a flow rate of 0.40 mL min^−1^ with an overall run time of 15 min.

MS/MS parameters were optimized by individual standard infusion at the concentration of 1 ng/μL in eluents stream. The precursor ions [M + NH_4_]^+^ with *m/z* = 1170.6 for the cereulide and *m*/*z* = 1176.7 for I.S. were selected and five product ions were chosen and monitored for the analyte (Table 4).

The following ESI source parameters were set for TSQ ENDURA: vaporization temperature (400 °C), spray voltage (3.0 kV), sheet gas pressure (20 arbitrary unit), auxiliary gas pressure (7 arbitrary unit), sweep gas pressure (0 arbitrary unit), capillary temperature (270 °C), collision gas pressure (argon, 2 mTorr), cycle time (1 s) and peak resolution on Q1 and Q3 (0.7 FWHM).

ESI source parameters for SCIEX QTRAP 6500+ were: TEM (350 °C), ion spray voltage (5500 V), Curtain gas (35 psi), Nebulizer gas (45 psi), Turbo gas (35 psi), CAD (medium), DP (10 V), EP (10 V), dwell time (30 ms) and peak resolution on Q1 and Q3 (1 FWHM).

ESI source parameters for SCIEX QTRAP 7500 were: TEM (450 °C), ion spray voltage (2000 V), Curtain gas (42 psi), Ion source gas 1 (15 psi), Ion source gas 2 (55 psi), CAD (9), EP (10 V), dwell time (30 ms) and peak resolution on Q1 and Q3 (1 FWHM).

### 2.5. Quantitative Analysis

Cereulide concentration in samples was determined using linear regression curves obtained from matrix-matched calibration standards. The relative response (cereulide peak area/internal standard peak area) was plotted against the corresponding analyte concentration in the matrix.

The instrumental limit of quantification (iLOQ) was initially established as the lowest concentration providing a signal-to-noise ratio (S/N) ≥ 10. Subsequently, the method limit of quantification (mLOQ) was validated through evaluation of trueness and precision in fortified matrix samples. The lowest concentration meeting the predefined acceptance criteria for recovery and repeatability was 0.025 μg/L. Therefore, 0.025 μg/L was established as the validated method LOQ and used as the lowest reportable quantification level.

Toxin concentrations were reported as μg/kg for food samples and as μg/L for reconstituted infant formula (ready-to-consume), in accordance with EFSA recommendations.

### 2.6. Evaluation of ISO 18465 Applicability

Response linearity was evaluated by preparing three different six-point calibration curves in the range 0.08–8 pg/μL, corresponding to cereulide final matrix concentrations of 1–100 μg/kg, all containing ^13^C_6_-cereulide at 20 μg/kg. The calibration curve was obtained by linear regression analysis of relative response (cereulide area/I.S. area) versus concentration.

The applicability of the standardized method was evaluated by comparing the precision reported in ISO 18465 with that obtained from our laboratory.

The studies of repeatability and recovery were carried out on 10 independent replicates of boiled rice fortified at 25 μg/kg, 10 independent replicates of pasta fortified at 25 μg/kg and 10 independent replicates of pasta fortified at LOQ level of 1 μg/kg.

### 2.7. In-House Method Validation for Infant Formula

The confirmatory quantitative LC-MS/MS method was fully validated considering the following parameters: response linearity, matrix effect, LOQ, precision (repeatability and within-laboratory reproducibility), trueness, ruggedness and analyte stability in standard solutions and final extracts.

Response linearity was evaluated by preparing three different six-point calibration curves in the range 0–100 pg/mL, corresponding to cereulide final matrix concentrations of 0–1 μg/L, all containing ^13^C_6_ -cereulide at 0.250 μg/L. The calibration curve was obtained by linear regression analysis of relative response (cereulide area/I.S. area) versus concentration.

The iLOQ was determined under optimized analytical conditions as the lowest concentration providing a signal-to-noise ratio (S/N) greater than 10. To establish the mLOQ, corresponding to the validated quantification level, a concentration of 0.025 μg/L was selected, representing approximately half of the maximum level (LM = 0.054 μg/L) which corresponds to the lower limit of the EFSA-established level of interest. This concentration was subsequently validated by assessing trueness and precision, confirming its suitability as the method LOQ.

To evaluate the matrix effect, process efficiency and recovery of extraction, a standard solution [A] was analyzed in the same analytical session with a sample spiked before extraction [C] and a sample spiked after extraction [B], according to the Matuszewski approach [36].

Trueness and precision were evaluated on the same blank infant formula sample spiked at four concentration levels (0.025, 0.050, 0.100 and 0.150 μg/L); six independent replicates for each level processed according to the procedure described in Section 2.3.2. These steps were repeated by different operators in different weeks.

Trueness was calculated for each sample using the equation below:

(1) Trueness (%) = (mean recovery−corrected concentration detected) × 100/spiked concentration

Subsequently the mean trueness was calculated for the six results at each concentration level.

Precision was calculated using an ANOVA function and expressed as the CV (%) of repeatability and within-laboratory reproducibility.

Ruggedness to minor changes was evaluated using the Youden approach [37] on a blank sample fortified at 0.100 μg/L. Five critical factors related to sample preparation were selected and evaluated at two levels, one above and one below the nominal value. For each factor, the effect of the variation was assessed using a *t*-test, while an F-test was applied to evaluate the overall variability associated with these experimental variations.

Final extract stability was tested for one week after storage at −20 °C.

Intermediate standard solution stability was tested by comparing standard work solutions made respectively from a new stock solution and a three-year-old stock solution stored at −20 °C.

## 3. Results and Discussion

### 3.1. Sample Preparation

#### 3.1.1. Food

Food sample preparation follows the ISO 18465 method with some minor changes. The sample preparation procedure was assessed using boiled rice and pasta. While pasta was easily and uniformly dispersed in the extraction solvent, rice formed a compact agglomerate, thereby limiting the contact surface between the sample and the solvent. Therefore, an additional homogenization step using an Ultra-Turrax was introduced after acetonitrile addition to ensure efficient sample dispersion and extraction. This observation is consistent with the challenges of extracting cereulide from starchy matrices, where incomplete solvent interaction can lead to underestimation of toxin levels.

The defatting step using 2 × 3 mL of n-heptane was introduced as mandatory for all samples to reduce the presence of interfering compounds, particularly in complex food products containing sauces, oils, vegetables, cheese, and infant formula, which represents the majority of the samples analyzed in routine official controls from outbreaks.

#### 3.1.2. Infant Formula

For infant formula, sample preparation is a critical step due to the complex and highly processed nature of this matrix.

According to the EFSA risk assessment of January 2026 [23], we reconstituted the infant formula powders using a powder-to-water ratio according to the manufacturer’s instructions (or a 1:8 ratio when not specified) to simulate the ready-to-consume product and ensure comparability with exposure assessments. In fact, direct extraction from dry powders using organic solvent can result in low cereulide recovery, as the toxin may remain entrapped within lipid–protein aggregates or encapsulated nutrient structures. Reconstitution was performed at 40 °C, as reported in previous studies [38]. After reconstitution, the extraction was carried out with acetonitrile, which also promotes protein precipitation, followed by a defatting step. The final extract was stable at −20 ° C for at least 1 week.

This easy and fast procedure allowed for the quantification of cereulide at the required concentration levels, unlike the method recently published by Zhou et al., 2026 [35], which involves an SPE purification step.

### 3.2. LC-MS/MS Analysis

For cereulide quantification by liquid chromatography coupled with tandem mass spectrometry (LC-MS/MS), we used the SIDA approach described in the ISO. The use of a stable isotope of cereulide (^13^C_6_-cereulide) as internal standard is able to counterbalance losses during extraction procedures and overcome matrix effect in the analysis.

The ISO 18465 method displayed the ionization and fragmentation parameters obtained using a Waters Micromass Quattro Premier system; the ionization and MS/MS parameters on Thermo TSQ Endura, SCIEX QTRAP 6500+ and SCIEX QTRAP 7500 system were optimized by infusing a standard solution of cereulide at 1 ng/μL.

The highest abundant ion in MS1 was at *m*/*z* = 1170.89, corresponding to the ammonium adduct [M + NH_4_]^+^.

Cereulide is a big molecule, and it can give rise to many MS/MS transitions: ISO 18465 Annex B shows over 30 product ions. Different fragmentation patterns are generated depending on the gas pressure in the collision cell of the second quadrupole. The same five more stable and high transitions were obtained by setting Collision Gas Pressure at 2 mTorr for Thermo TSQ Endura, CAD “Medium” for SCIEX QTRAP 6500+ and CAD 9 for SCIEX QTRAP 7500.

The identification of cereulide was achieved through a total of 9.5 identification points according to Commission Implementing Regulation (EU) 2021/808 (CIR) criteria [39]: 1 point from chromatographic separation, 1 point from the precursor ion detected in the MS/MS experiment, and 1.5 points from each of the five product ions monitored. Considering the legal consequences in case of a non-compliant sample, this criterion guarantees the unambiguous confirmation of the cereulide presence in the meal, without false-positive results.

Several HPLC columns as well as different organic mobile phases (methanol, acetonitrile) were tested for chromatographic conditions (Table 5).

**Figure 2:**
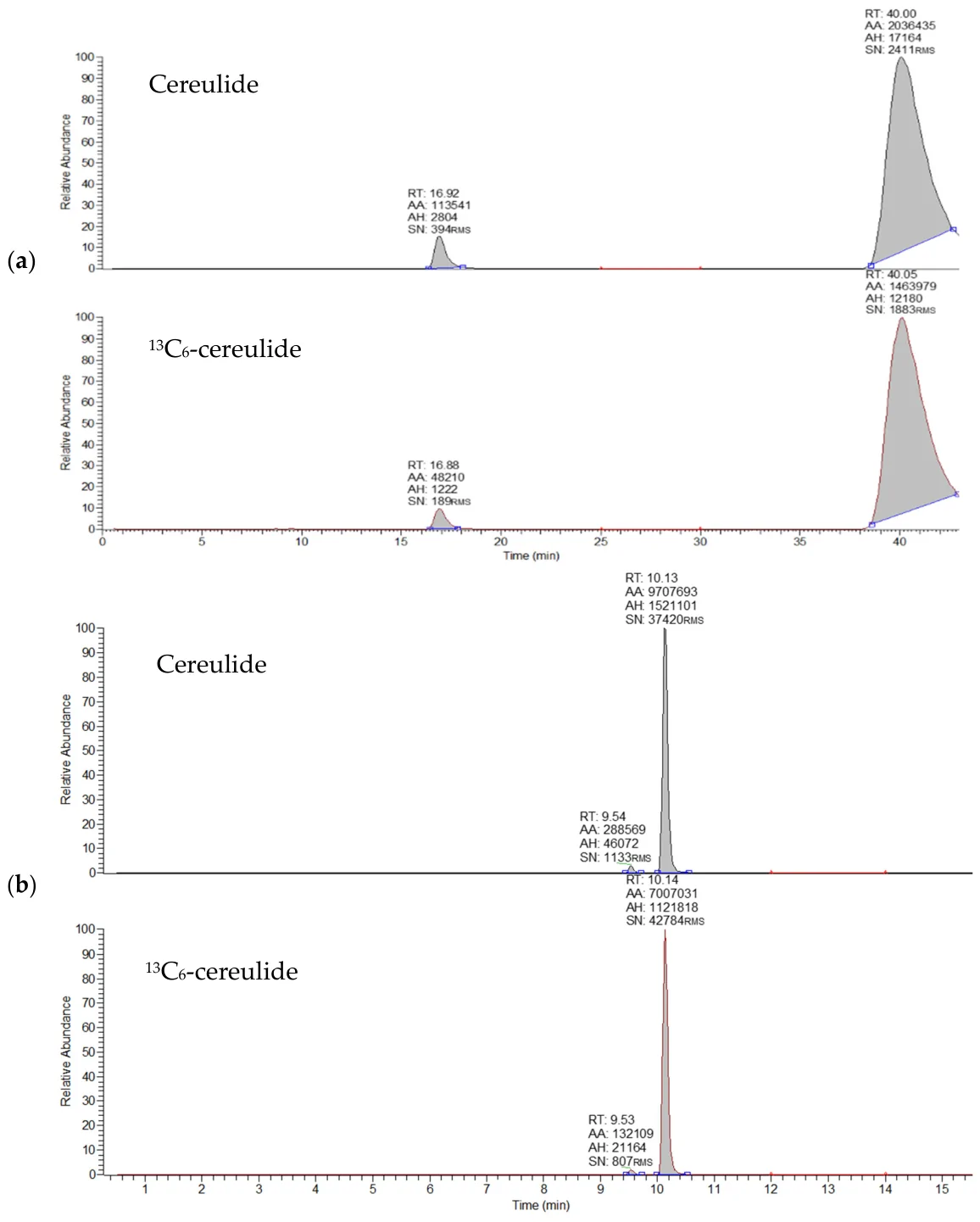
MRM chromatogram of cereulide and ^13^C_6_-cereulide using acetonitrile (**a**) or methanol (**b**) as organic mobile phase.

The columns with a low degree of end-capping showed a very broad peak, probably due to the interactions of amide and ester groups of the molecule with the free silanols; only the columns with complete end-capping were found to be exempt from this problem. The X-Select HSS T3 C18 column (Waters) with methanol as eluent gave very good results in terms of retention time, S/N ratio, peak shape and symmetry, while using acetonitrile, cereulide and I.S. eluated after 40 min with a large peak and tailing peaks (Figure 2).

Using a shorter column (100 mm), no loss of peak shape and high resolution was detected. Moreover, the run time was reduced from 25 to 15 min, with an important implication for routine total batch analysis time: this was selected as the best option.

The same instrumental conditions were used for infant formula analysis, but, due to the lower concentration levels, only the SCIEX QTRAP 7500 was suitable for this application. The QTRAP 7500 demonstrated superior sensitivity, achieving an mLOQ of 0.025 µg/L, whereas neither the QTRAP 6500 nor Endura instruments were able to achieve the same concentration level necessary for infant formula analysis.

**Figure 3:**
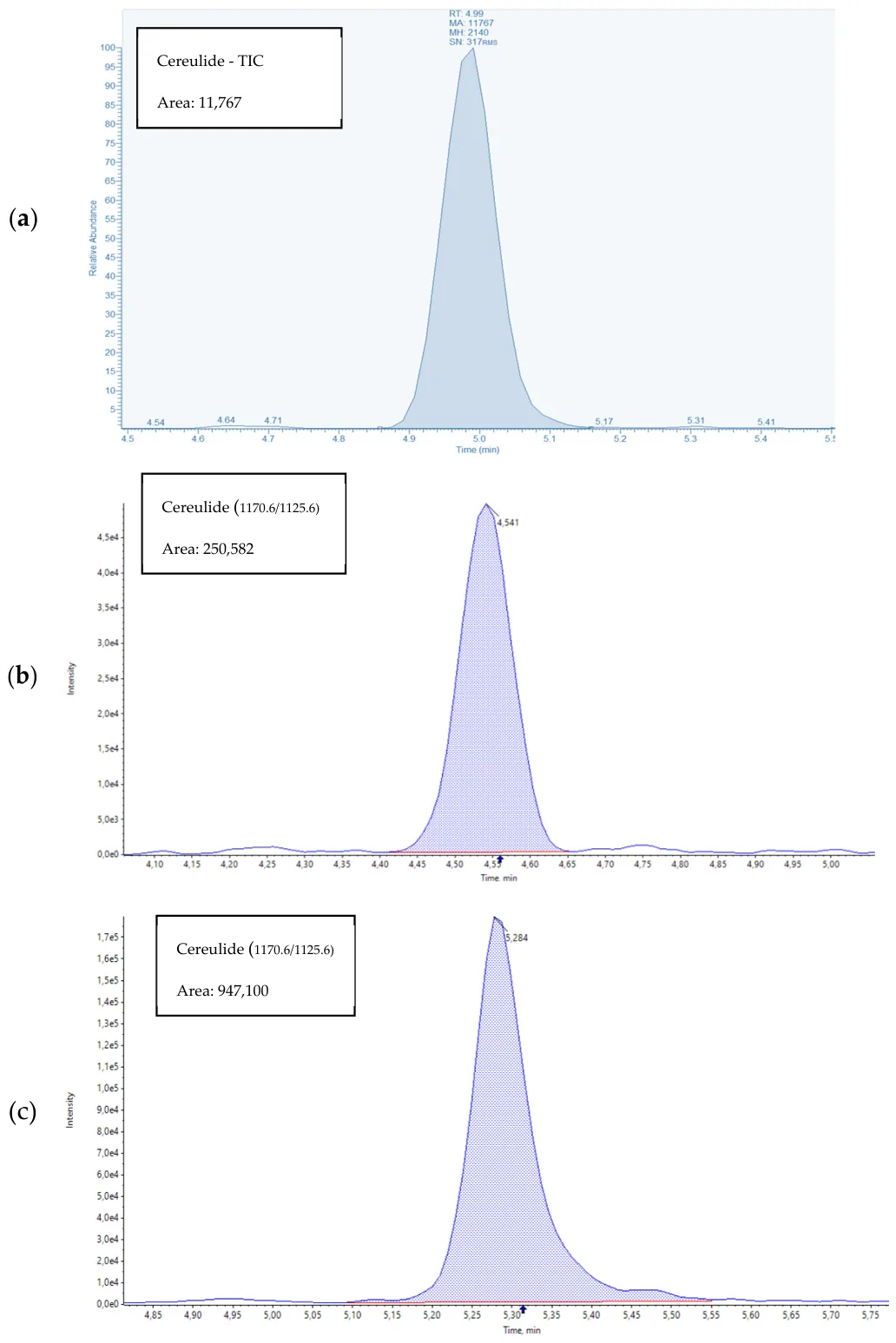
MRM chromatogram of a sample spiked with cereulide at 1 μg/kg on Thermo TSQ Endura (**a**), SCIEX QTRAP 6500+ (**b**) and SCIEX QTRAP 7500 (**c**).

Indeed, testing different samples spiked with cereulide at 1 μg/kg using all LC-MS/MS systems and comparing the mean areas, the Sciex QTRAP 7500 showed an approximately five-fold higher response than the SCIEX QTRAP 6500+ system (Figure 3).

### 3.3. Evaluation of ISO 18465 Applicability

Response linearity was verified in the range 0.08–8 pg/μL, corresponding to cereulide final matrix concentrations of 1–100 μg/kg. Solutions of cereulide (0.08–0.4–0.8–1.6–4.0–8.0 pg/μL) in acetonitrile, all containing ^13^C_6_-cereulide at 1.7 pg/μL, were injected three times. The calibration curve was obtained by linear regression analysis of relative response (cereulide area/I.S. area) versus concentration. The fitted calibration curve was linear in the tested concentration range, with R^2^ = 0.9999.

ISO 18465 identifies repeatability and recovery as the primary validation parameters. The applicability of the standardized method was assessed by comparing the precision obtained in experimental tests and by confirming, through secondary validation, that the repeatability standard deviation complied with the specified acceptance criteria.

**Figure 4:**
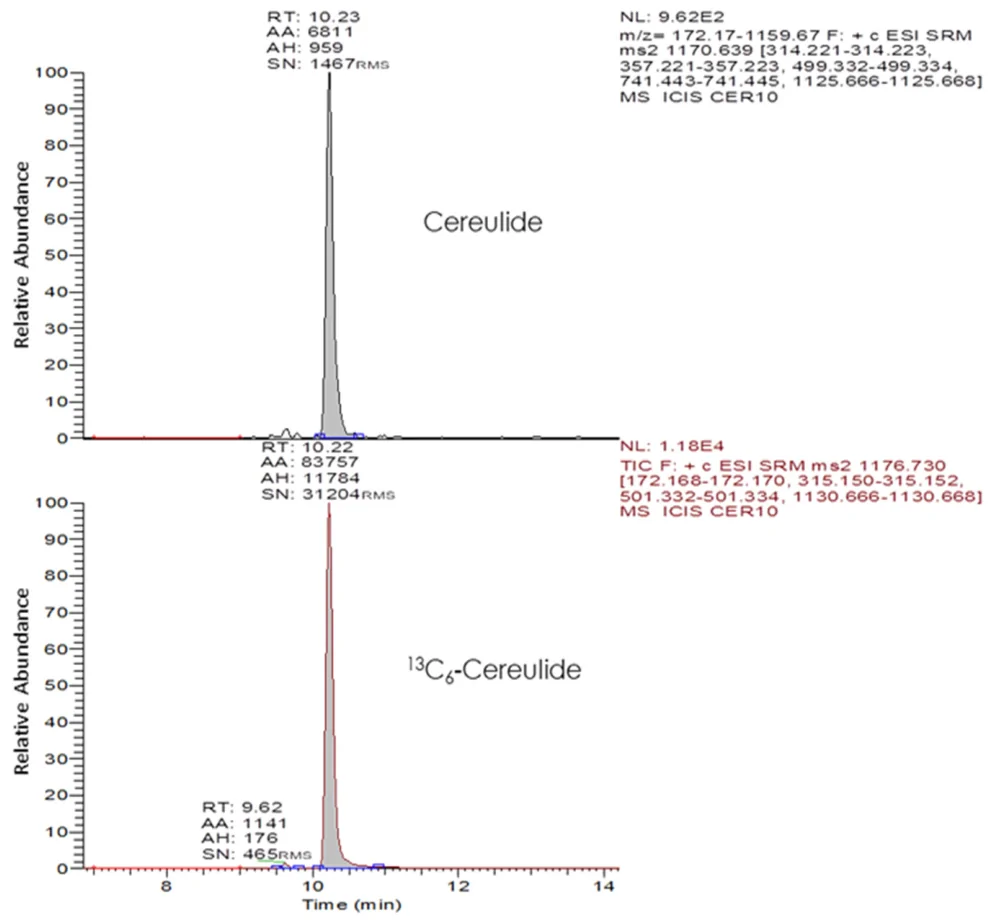
Chromatogram of rice spiked at 1 µg/kg registered on Thermo TSQ Endura system.

The studies of repeatability and recovery were carried out on 10 samples of boiled rice and 10 samples of pasta all fortified at 25 μg/kg. Repeatability at LOQ level of 1 μg/kg (Figure 4) was evaluated on dishes prepared with different dressings; s_r_ and coefficient of variation (CV_r_ %), were calculated for each preparation. Mean recoveries for samples fortified in the 25 μg/kg range between 90 and 100% while at LOQ level were little higher (116%) as shown in Table 6.

A secondary validation can be applied if the following equation is satisfied:

(2) A ≤ s_r_ / σ_r_ ≤ B

Value A and B are tabulated [40] and represent the minimum (A) and maximum (B) confidence limits of the ratio, s_r_/σ_r_, as function of the degrees of freedom. In Table 7 the obtained values of s_r_ (0.46) were compared to those reported in the ISO 18465 (σ_r_ = 0.80) at the corresponding levels, ν = *n* − 1, where *n* is the number of tests; s_r_ and σ_r_ indicate respectively the repeatability standard deviation of laboratory and the one reported in the ISO. The results met the requirements expressed in the above Equation (3).

The presence of interferences was evaluated on meals containing rice or pasta with meat, vegetables, and other condiments. No interfering signal was detected in any of the samples analyzed.

The same complex meals were analyzed also after spiking with cereulide at 10 μg/kg; no differences in response due to variability of the meal composition were found, thus results show a mean recovery of 100% with a dispersion among matrixes less than 2.5%.

The SIDA approach based on the use of labeled cereulide allowed good recovery correction and minimized the matrix effect, justifying the use of a calibration curve in solvent for quantitative analyses.

This evidence was further confirmed in routine analysis of more than 300 samples composed of different types of meals, in which a perfect correspondence between the I.S. in the control (starchy food) and in the sample was observed, thus indicating that no matrix effect can be detected. This outcome makes the method suitable for use both in food inspection and in cases of suspected foodborne disease when it is necessary to analyze not only starchy foods but the most varied foods from canteens, restaurants, delicatessen, etc.

Laboratory trueness was verified using the new CRM cooked rice provided by JRC of Geel, with a found value of 11.1 μg/kg (assigned value = 11.3 ± 0.8 μg/kg).

### 3.4. In-House Method Validation for Infant Formula

The confirmatory quantitative LC-MS/MS method was fully validated considering the following parameters: response linearity, matrix effect (ME), LOQ, precision (repeatability and within-laboratory reproducibility), trueness, ruggedness and analyte stability in standard solutions and final extracts (Table 8).

The response was linear in the range 1–100 pg/mL in solvent, corresponding to 0.010–1.000 μg/L in matrix: linear regression equation was y = 7.0253x + 0.0046, with R^2^ = 1.000.

The matrix effect was calculated using the following equation:

(3) ME % = B/A × 100

where [B] = absolute peak area of sample spiked after extraction, and

[A] = absolute peak area of standard solution

The matrix effect was 96% in infant formulae and 89% in follow-on formulae, indicating a low ion suppression.

Extraction recovery was calculated using the following equation:

(4) RE % = C/B × 100

where [C] = absolute peak area of sample spiked before extraction, and

[B] = absolute peak area of sample spiked after extraction

Extraction recovery was 88% in infant formulae and 92% in follow-on formulae.

Process efficiency was calculated using the following equation:

(5) PE % = C/A × 100

where [C] = absolute peak area of sample spiked before extraction, and

[A] = absolute peak area of standard solution

Process efficiency was 84% in infant formulae and 81% in follow-on formulae.

The SIDA approach improves the accuracy of cereulide quantification by using the labeled I.S. ^13^C_6_-cereulide. Since the labeled I.S. has physiochemical properties very similar to the analyte, it undergoes similar losses during sample preparation and is similarly affected by matrix induced ion suppression. Therefore, quantification based on the analyte to I.S. response ratio compensates for these variations, reducing their impact on the result.

Indeed, during the validation, trueness calculated ranged between 102 and 109%.

**Figure 5:**
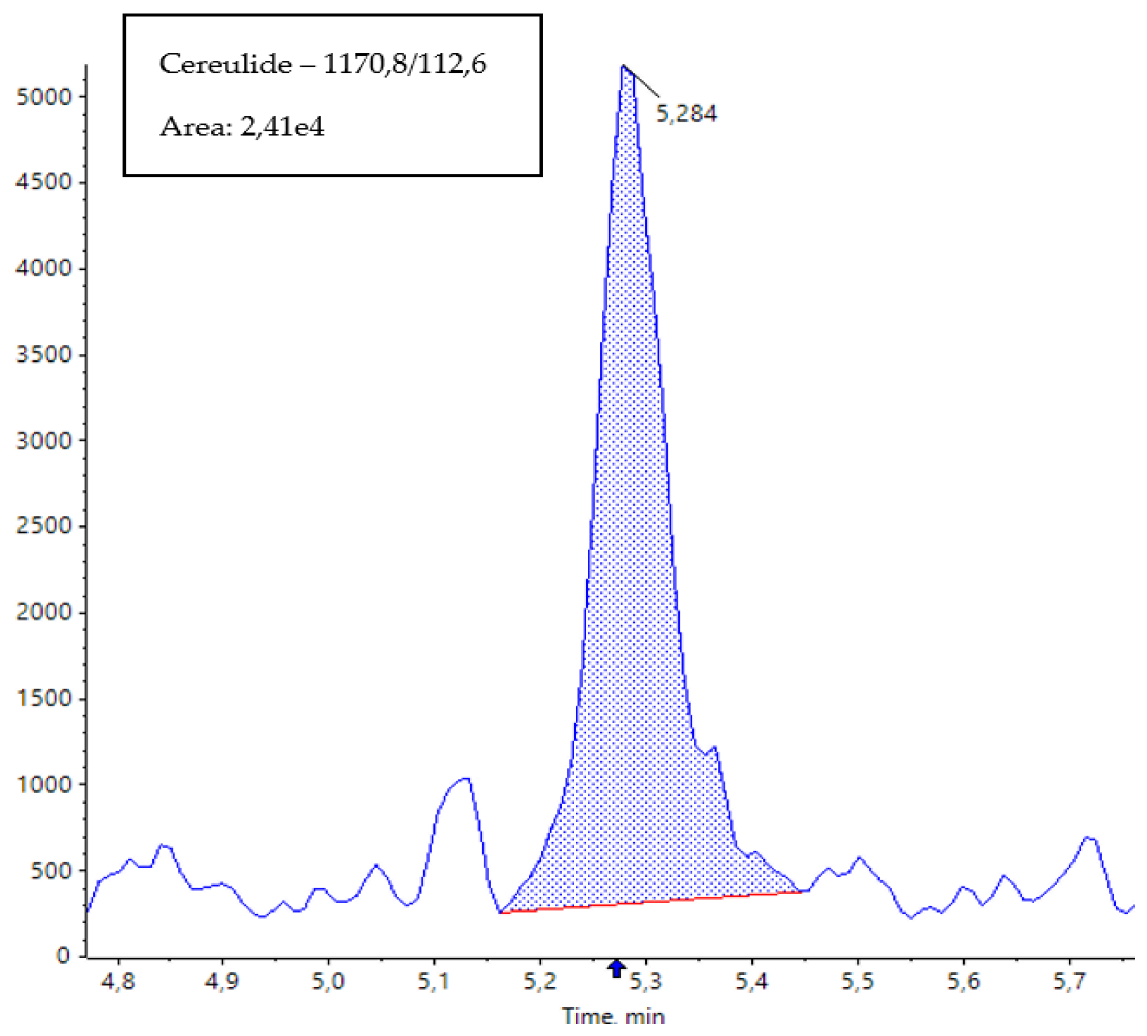
Chromatogram of infant formula spiked with cereulide at 0.025 µg/L (mLOQ) registered on SCIEX QTRAP 7500 system.

The instrumental limit of quantification (iLOQ) was below 0.010 μg/L in matrix, as the lowest calibration level (1 pg/mL) produced a signal-to-noise ratio (S/N) of approximately 50, well above the acceptance criterion of S/N ≥ 10. The method limit of quantification (mLOQ), corresponding to the validated quantification level, was established at 0.025 μg/L, a concentration selected as approximately half of the lower EFSA-established level of interest (0.054 μg/L). This level was subsequently validated by assessing ion ratio compliance, trueness, and precision, confirming its suitability for reliable quantitative determination (Figure 5).

This value is half the EFSA threshold for cereulide in reconstituted infant formula (0.054 μg/L), demonstrating that the method is suitable for official controls.

Given the absence of specific regulatory requirements for the validation of analytical methods intended for the determination of bacterial toxins, the performance criteria established in Commission Implementing Regulation (CIR) (EU) 2021/808 for the analysis of residues of pharmacologically active substances were adopted as a reference framework. This Regulation replaced Decision 2002/657/EC, which is cited in ISO 18465 as a reference document for method validation.

According to the CIR 808/2021 [39] requirements for confirmatory methods, trueness at concentration level below 1 μg/kg should be in the tolerance range from 50% to 120%: our values ranged from 102% to 109%, so they can be considered satisfactory.

About method precision, the within-laboratory reproducibility CV% obtained was ≤13%, according to the maximum CV% of 30 established by CIR 808/21.

Ruggedness, evaluated with the Youden approach [37], found that the method was robust to minor changes. All investigated factors showed no significant influence since each calculated t-values were below the tabulated critical value. Moreover, the calculated F-value was lower than the tabulated critical value, indicating that the introduced variations did not significantly affect method variability.

Final extracts were stable for almost one week after storage at −20 °C.

Intermediate standard solution stability was satisfactory (within ± 5%) for almost three years after storage at −20 °C.

The expanded relative measurement uncertainty, estimated using the top-down approach, was 23%. This value demonstrates good method performance and is substantially lower than the indicative maximum uncertainty of 50% reported in Commission Implementing Regulation (EU) 2023/2782 [41] for mycotoxin analysis in food. Although this regulation is not specifically applied to cereulide in infant formula, the comparison provides a useful benchmark, supporting the fitness-for-purpose of the proposed method for quantitative determination of cereulide at concentrations relevant to exposure assessment.

### 3.5. Quality Control Assurance

In each analytical batch, a blank control matrix and the same control matrix spiked with cereulide at mLOQ level were assessed: in six years of routinary controls no false-negative or false-positive results were found.

The control chart with cereulide amount found in spiked control food showed that the method performances are stable over time.

The analysis of contaminated rice as a CRM obtained by the JRC of Geel confirmed the trueness evaluated during the validation.

### 3.6. Six-Year Survey

Between 2019 and 2024, a total of 332 food samples from suspected foodborne outbreaks were collected throughout Italy and delivered to Laboratory of the Istituto Zooprofilattico Sperimentale del Piemonte, Liguria and Valle d’Aosta (IZSPLV) in Turin for quantitative determination of cereulide.

In five samples from different foodborne outbreaks, a positive result was obtained. The first positive meal was a homemade salad with potatoes, shrimps and squid collected after the rise in different food poisoning symptoms such as vomit, diarrhea, shivers and fever among the family members. The meal was left at room temperature overnight and then stored in the fridge until its consumption the following evening. The analysis showed a concentration of cereulide of 1.05 µg/kg.

In the second case, 532 µg/kg of cereulide were found in a sample of Italian crepes seized from a bistro after a suspected foodborne disease.

Two different samples of poke bowls (rice with courgettes, carrots, tuna, salmon and tomatoes) contained 13 µg/kg and 25 µg/kg of cereulide.

Finally, a rice-based croquette (a typical Sicilian street food named ‘arancino’) was taken from a delicatessen following a foodborne intoxication. The toxin in the food was quantified at 2000 µg/kg. To compare the obtained data to those reported in the literature on foodborne outbreaks, we considered that an arancino weighs about 150–200 g, so the total amount of cereulide contained in it was equal to 350–400 µg. An average dose of cereulide of 8.7 μg/kg body weight was obtained, assuming that the food was eaten by a 60 kg person. This data agrees with the values reported in the literature, which estimate the emetic dose of cereulide at 8–10 μg/kg [17,18].

Looking into the composition of the positive samples, 20% contained potatoes, 60% were made with rice and 20% were starch-rich foods. The composition of the five positive samples suggested a link between the toxin’s presence and a high starch content in food, as reported in the literature [21,22].

## 4. Conclusions

The presented work integrates three complementary components that collectively address current challenges in cereulide surveillance and outbreak investigations. First, a secondary validation of the ISO 18465:2017 method was performed to verify its applicability and robustness under routine laboratory conditions. Second, a dedicated validation study was conducted for infant formula, a matrix not covered by ISO 18465 and characterized by specific analytical challenges, including pronounced matrix effects and stringent sensitivity requirements. Third, the validated methods were applied to the retrospective analysis of samples collected during foodborne outbreak investigations, providing real-world evidence of their suitability for official control activities.

The connection among these components lies in the establishment of a comprehensive analytical framework for cereulide detection across diverse food matrices. Beyond confirming the performance of ISO 18465, this study extends its applicability to infant formula through the validation of a fit-for-purpose method and demonstrates the practical value of both approaches in outbreak surveillance. In contrast to previous studies that focused either on method standardization for starch-rich foods or on the development of highly specialized procedures for infant formula, the present work offers an integrated approach linking method validation, matrix extension, and routine application to public health investigations.

The results obtained in this study demonstrate that the modified ISO 18465 method is robust, sensitive, and suitable for the determination of cereulide in a wide variety of food matrices.

From an analytical perspective, the method can be easily applied to different LC-MS/MS for food matrices, while only a high performing instrument like QTRAP 7500 can reach the sensibility required for infant formulae.

The SIDA approach allowed for a good recovery correction and justified the use of a calibration curve in solvent, thus simplifying the analytical workflow. Moreover, the use of labeled internal standard minimizes the matrix effect and makes the method suitable in cases of suspected foodborne disease, when it is necessary to analyze the most varied foods from canteens, restaurants, delicatessen, etc.

The easy and fast extraction procedure validated to quantify the cereulide at the concentration levels required for infant formula without using SPE purification step avoids time being wasted during routine analysis.

The six-year survey confirms the suitability of the validated method for cereulide monitoring in samples collected during suspected foodborne outbreaks. While the study was not intended to estimate cereulide prevalence in Italian foods, the concentrations detected in positive samples were consistent with toxic levels reported in the literature. The identification of cereulide in starch-rich foods agrees with previously reported observations, although the limited number of positive samples precludes establishing a statistical association.

## Figures and Tables

**Table 1:** Intermediate standard solution concentrations.

Solution	Concentration Level (ng/μL)
Cereulide–B	1.0000
Cereulide–C	0.1000
Cereulide–D	0.0100
Cereulide–E	0.0002
^13^C_6_-cereulide–C	0.1000
^13^C_6_-cereulide–D	0.0100
^13^C_6_-cereulide–E	0.0005

**Table 2:** Calibration standard solution concentrations for food analysis.

Level	Cereulide–D Volume (μL)	^13^C_6_-Cereulide–D Volume (μL)	Acetonitrile Volume (μL)	Cereulide Concentration Corresponding in Matrix (μg/kg)
0	0	200	1000	0
1	10	200	990	1
2	50	200	950	5
3	100	200	900	10
4	200	200	800	20
5	500	200	500	50
6	1000	200	0	100

**Table 3:** Calibration standard solution concentrations for infant formula analysis.

Level	Cereulide–E Volume (μL)	^13^C_6_-Cereulide–E Volume (μL)	Final Volume (mL)	^13^C_6_-Cereulide Concentration Corresponding in Matrix (μg/L)	Cereulide Concentration Corresponding in Matrix (μg/L)
0	0	500	10	0.250	0.000
1	50	500	10	0.250	0.010
2	100	500	10	0.250	0.020
3	250	500	10	0.250	0.050
4	500	500	10	0.250	0.100
5	2500	500	10	0.250	0.500
6	5000	500	10	0.250	1.000

**Table 4:** MRM optimized parameters on AB SCIEX instruments.

Analyte	Precursor Ion (*m*/*z*)	Product Ion (*m*/*z*)	Collision Energy (V)	CXP (V)
Cereulide	1170.6	1125.6 (a*)	54	20
		357.2	85	
		499.3	74	
		741.4	67	
		314.2	92	
Cereulide -^13^C_6_	1176.7	1130.6 (a*)	55	20
		944.5	62	
		744.5	65	
		501.5	74	
		315.3	90	

a* = quantifier.

**Table 5:** Evaluation of column performances.

Column	Length × i.d. (mm)	Particle Size (μm)	Peak Width at 50% (min)	S/N
Waters X-Select HSS T3	100 × 3.0	2.5	0.11	3170 (best)
Waters X-Select HSS T3	150 × 3.0	2.5	0.12	3000
Waters Acquity HSS CN	100 × 2.1	2.5	0.15	1500
Phenomenex Synergi MAX	150 × 2.1	4.0	0.27	1000
Phenomenex Synergi FUSION	150 × 2.1	4.0	0.50	200
Phenomenex Luna C8	150 × 4.6	4.0	0.45	320
Waters X-Terra C18	150 × 2.1	3.5	broad	low response
Phenomenex Kinetex Phenyl-Hexyl	75 × 2.1	2.6	broad	low response

**Table 6:** Results of secondary validation.

Levels	Cooked Rice	Cooked Pasta	Cooked Rice + Pasta	Meals
	25 µg/kg	25 µg/kg	1 µg/kg (LOQ)	10 µg/kg
Number of samples	10	10	12	7
s_r_ (µg/kg)	0.46	0.89	0.05	0.22
CV_r_%	1.84	3.56	4.81	2.50
Repeatability limit (µg/kg)	1.5	2.8	0.15	0.78
Recovery (%)	96	91	116	100

**Table 7:** Comparison between the precisions of the laboratories.

ν = (n − 1)	s_r_	σ_r_	Ratio s_r_/σ_r_	A	B
9	0.46	0.80	0.575	0.548	1.454

**Table 8:** Validation results for infant formula.

Spiking Level (μg/L)	0.025 (mLOQ)	0.050	0.100	0.150
Repeatability limit “r” (μg/L)	0.011	0.011	0.026	0.031
Repeatability CV (%)	12	6.1	7.5	6.0
Within-laboratory reproducibility CV (%)	13	6.5	8.0	13
Trueness %	109	108	106	102

## Data Availability

The data presented in this study are available on request from the corresponding author due to privacy and ethical restrictions.

## Abbreviations

The following abbreviations are used in this manuscript:

ARA	Arachidonic Acid
ARfD	Acute Reference Dose
CFU	Colony-Forming Unit
CIR	Commission Implementing Regulation
CRM	Certified Reference Material
EFSA	European Food Safety Authority
FBOs	Food-Borne Outbreaks
ISO	International Organization for Standardization
JRC	Joint Research Center
LC-MS/MS	Liquid Chromatography with Tandem Mass Spectrometry
LOQ	Limit Of Quantification
ME	Matrix Effect
ML	Maximum Limit
MRM	Multiple-Reaction Monitoring
RSD	Relative Standard Deviation
SIDA	Stable Isotope Dilution Analysis
SPE	Solid-Phase Extraction
UHPLC	Ultra-High-Performance Liquid Chromatography
